# Virulence Determinants and Genetic Diversity of *Yersinia* Species Isolated from Retail Meat

**DOI:** 10.3390/pathogens11010037

**Published:** 2021-12-29

**Authors:** Margarita Terentjeva, Juris Ķibilds, Irēna Meistere, Silva Gradovska, Laura Alksne, Madara Streikiša, Jevgēnija Ošmjana, Olga Valciņa

**Affiliations:** 1 Institute of Food and Environmental Hygiene, Faculty of Veterinary Medicine, Latvia University of Life Sciences and Technologies, LV-3004 Jelgava, Latvia; 2 Institute of Food Safety, Animal Health and Environment BIOR, LV-1076 Riga, Latvia; Juris.Kibilds@bior.lv (J.Ķ.); Irena.Meistere@bior.lv (I.M.); Silva.Gradovska@bior.lv (S.G.); Laura.Alksne@bior.lv (L.A.); Madara.Streikisa@bior.lv (M.S.); Jevgenija.Osmjana@bior.lv (J.O.); Olga.Valcina@bior.lv (O.V.)

**Keywords:** *Yersinia enterocolitica*, prevalence, antimicrobial resistance, pork, WGS, cgMLST, virulence factors, Latvia

## Abstract

*Yersinia enterocolitica* is an important foodborne pathogen, and the determination of its virulence factors and genetic diversity within the food chain could help understand the epidemiology of yersiniosis. The aim of the present study was to detect the prevalence, and characterize the virulence determinants and genetic diversity, of *Yersinia* species isolated from meat. A total of 330 samples of retailed beef (n = 150) and pork (n = 180) in Latvia were investigated with culture and molecular methods. Whole genome sequencing (WGS) was applied for the detection of virulence and genetic diversity. The antimicrobial resistance of pathogenic *Y. enterocolitica* isolates was detected in accordance with EUCAST. *Yersinia* species were isolated from 24% (79/330) of meats, and the prevalence of *Y. enterocolitica* in pork (24%, 44/180) was significantly higher (*p* < 0.05) than in beef (13%, 19/150). *Y. enterocolitica* pathogenic bioserovars 2/O:9 and 4/O:3 were isolated from pork samples (3%, 6/180). Only resistance to ampicillin was confirmed in *Y. enterocolitica* 4/O:3 and 2/O:9 isolates, but not in other antimicrobials. Major virulence determinants, including *ail*, *inv*, *virF*, *ystA* and *myfA*, were confirmed with WGS in *Y. enterocolitica* 2/O:9 and 4/O:3. MLST typing revealed 15 STs (sequence types) of *Y. enterocolitica* with ST12 and ST18, which were associated with pathogenic bioserovars. For *Y. enterocolitica* 1A, *Y. kristensenii*, *Y. intermedia* and *Y. frederiksenii*, novel STs were registered (ST680-688). The presence of virulence genes and genetic characteristics of certain *Y. enterocolitica* STs confirm the common knowledge that pork could be an important source of pathogenic *Yersinia*.

## 1. Introduction

The *Yersinia* genus currently consists of 28 species, of which three are human pathogenic, while others are considered as non-pathogenic, *Yersinia*-like microorganisms [1,2]. Pathogenic *Yersinia enterocolitica* and *Yersinia pseudotuberculosis* are reported to cause yersiniosis, which is a zoonotic foodborne infection characterized by gastrointestinal manifestations, and post-infection sequelas, such as reactive arthritis or erythema nodosum [3,4]. Yersiniosis is reported to be the fourth most common bacterial zoonosis within the European Union [5].

*Y. enterocolitica* is a very heterogeneous species and is divided into six biotypes and various serogroups with different bioserovars showing distinctive virulence properties, hosts and geographical distribution [1]. *Y. enterocolitica* biotype 1A is non-pathogenic since it lacks classical virulence markers, which are important for the invasion of the human host and survival in the organisms [4,6,7]. Non-pathogenic *Yersinia* and *Y. enterocolitica* are widely distributed in the environment, animals and food and were isolated from clinical patients [8,9]. *Y. enterocolitica* biotypes 1B-5 are pathogenic, and bioserovars of 2/O:5,27, 2/O:9, 3/O:3 and 4/O:3 were recorded in clinical cases in Europe [1]. 

Pathogenic *Y. enterocolitica* were reported to be present in animal hosts, although they were rarely associated with meats other than pork [10,11]. Pigs are suspected to be important carriers of pathogenic *Yersinia*, and the contamination of pork may occur during slaughter as a result of cross-contamination [12]. Pathogenic *Yersinia* were identified in pig carcasses at the slaughterhouses, meat processing environment and at the retail [10,13]. Pathogenic *Y. enterocolitica* has been frequently isolated from pork—retail cuts, minced pork, offal and pork sausages—with the majority of isolated strains belonging to the same bioserotypes that were identified in pigs—4/O:3 [13,14,15,16,17]. Undercooked pork meat has been significantly associated with sporadic yersiniosis cases, but the genetic similarity between the human and porcine isolates indicates transmission through the pork production chain [10,18,19]. Thus, studies on the prevalence of pathogenic *Yersinia* species in meats are important for the recognition of foodborne transmission and the assessment of the distribution within the food chain. 

Pathogenic *Y. enterocolitica* carry both chromosomal (*ail*, *invA* and *ystA*) and plasmid-borne (plasmid of *Yersinia* virulence, pYV) genes, e.g., *yadA* and *virF*, which are required for full virulence [20]. The present, widely recognized methodology to differentiate between non-pathogenic and pathogenic *Yersinia* species mostly relies on the detection of the *ail* gene (adhesion and invasion locus) [21]. Notwithstanding, the presence of virulence markers, including the *ail* gene, was reported in non-pathogenic *Yersinia* species and *Y. enterocolitica* 1A isolates [22]. Therefore, the characterization of virulence factors in *Yersinia* isolates is important for an understanding of the pathogenicity potential of the *Yersinia* species as different *Y. enterocolitica* virulotypes and virulence traits could be established in *Yersinia* species [19,23]. 

New advances in food safety research show that the application of novel microbial typing methods as whole genome sequencing (WGS) may contribute to the knowledge on the virulence and phylogenetic relationships of the microbial isolates of public health importance [24]. The highly discriminatory approach provided by the WGS is crucial for surveillance, epidemiological investigations of yersiniosis and the virulence assessment of *Yersinia* species and may provide a new insight into the epidemiology of *Yersinia* in the food chain [25,26]). 

Since there is limited information on the virulence characteristics and genetic diversity of the *Yersinia* species in meat, the aim of the present study was to investigate the prevalence, characterize virulence factors and describe the genetic diversity of *Yersinia* isolates recovered from retail meats.

## 2. Results

### 2.1. Prevalence of Yersinia *spp.* and Pathogenic Yersinia Enterocolitica Bioserovars in Meats

The overall prevalence of *Yersinia* spp. in meats was 24% (79/330). One to three *Yersinia* spp. were found in one investigated sample. The highest number of *Yersinia* was found in pork cuts with five isolated species: *Y. enterocolitica* (23%, 36/160), *Y. intermedia* (3%, 4/160), *Y. kristensenii* (1%, 1/160) and *Y. frederiksenii* (2%, 1/160). The lowest diversity of the *Yersinia* species was recovered from beef, where 19% (13/150) of *Y. enterocolitica*- and 4% (6/150) of *Y. intermedia*-positive samples were identified. The prevalence of *Y. enterocolitica* in meats was higher than the prevalence of other *Yersinia* species (*p* < 0.05) (Table 1). 

A significantly higher prevalence of *Y. enterocolitica* of 24% (44/180) was identified in pork in comparison to 13% (19/150) (*p* < 0.05) in beef. In addition to meat categories, the highest prevalence of the *Yersinia* species of 55% (6/11) and *Y. enterocolitica* of 45% (5/11) was detected in offal, while the lowest was detected in beef cuts of 16% (24/150) and 19% (13/150), respectively (Table 1).

Out of the 63 *Y. enterocolitica*-positive samples, five belonged to bioserovar 4/O:3, one to 2/O: 9 and 57 to biotype 1A. The presence of the *ail* gene was confirmed in all *Y. enterocolitica* 4/O:3 and 2/O:9 isolates with qPCR (Supplementary Table S3). 

### 2.2. Antimicrobial Resistance in Yersinia enterocolitica 2/O:9 and 4/O:3 Isolates

Antimicrobial resistance against ampicillin was identified in 100% of *Y. enterocolitica* 4/O:3 and 2/O:9. All *Y. enterocolitica* 4/O:3 and 2/O:9 isolates were susceptible to cefotaxime, ceftazidime, ciprofloxacin, chloramphenicol, colistin, gentamicin, meropenem, tetracycline and trimethoprim (Table 2). Differences between the antimicrobial resistance pattern of *Y. enterocolitica* of biotypes 4/O:3 and 2/O:9 were not found. 

### 2.3. Genetic Diversity and Virulence of Yersinia Isolates

MLST sequence types were identified for all sequenced isolates. Among these, nine novel STs were identified and registered in Enterobase (ST680-ST688). Most of the novel STs were from non-*enterocolitica* species.

Based on WGS data analysis, 15 STs of *Y. enterocolitica* were identified where all pathogenic 4/O:3 isolates belonged to ST18 but all 2/O:9 isolates belonged to ST12. One *Y. enterocolitica* 4/O:3 was excluded from WGS analysis due to contamination (Supplementary Table S3). Non-pathogenic *Y. enterocolitica* belonged to 13 STs, and one to two isolates of each ST were recovered (Table 3). Each isolate of *Y. frederiksenii*, *Y. intermedia* and *Y. kristensenii* represented one ST (Table 3). All but one isolates originated from pork, while ST137 was identified in beef. 

**Figure 1:**
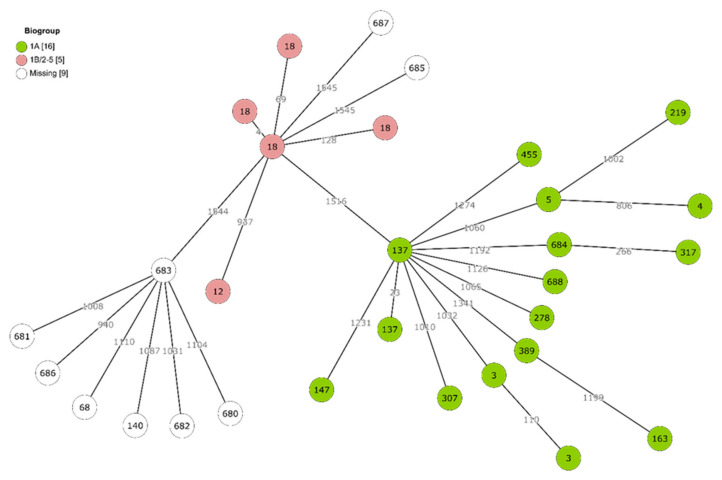
Minimum spanning tree of *Yersinia* cgMLST profiles: Branch lengths are drawn in log scale. For each node, MLST sequence type number is indicated. Coloured nodes represent virulent or non-virulent *Y. enterocolitica* biotypes that were determined based on presence of *ail*, *inv*, *ystA* and *ystB* genes. Uncoloured nodes represent non-*enterocolitica* species for which this biotype determination was not applicable.

The genetic structure of the *Yersinia* population was explored in more detail with the whole-genome multilocus sequence typing (cgMLST) approach, which is based on 1553 loci (Figure 1). On average, >1000 allelic differences separated individual isolates. No dense clusters of genotypes could be observed. Instead, they appeared to be scattered with large distances between them, with exceptions when multiple isolates shared the same ST (e.g., multiple strains representing ST3, ST18 and ST137).

The most common virulence determinants in all *Yersinia* species were *ymoA* (100%) followed by *fepD* and *fes*. All *Y. enterocolitica* harboured *hreP*, *inv*, *myfB*, *myfC*, *sat* and *ymoA* virulence genes. Out of pathogenic *Y. enterocolitica*, ST18 was the only *fepD*- and *fes*-negative ST, but shared *ail*, *hreP*, *inv*, *myfA*, *myfB*, *myfC*, *sat*, *virF*, *yadA*, *ymoA* and *ystA* (Table 4, Supplementary Table S1). *Y. enterocolitica* ST12 contained all virulence factors of ST18, with the exception of *yadA*, and was *fepD* and *fes* positive. 

Based on the combined presence or absence of *ail*, *inv*, *ystA* and *ystB* virulence genes, *Y. enterocolitica* strains could be classified as virulent or non-virulent biotypes. Only two STs (ST12 and ST18) were represented among the virulent biotypes 1B/2–5. Many more isolates and a wide range of STs were classified as the non-virulent 1A biotype (Figure 1).

Limited diversity in virulence was observed between *Y. enterocolitica* 1A STs and was related to the presence of *myfA* in ST 317, 389, 684 and 688, and the absence of *fepD* and *fes* in ST688.

All *Yersinia* species other than *Y. enterocolitica* shared *fepD* and *ymoA*, and all were lacking *ail*, *inv*, *myfA*, *myfB*, *myfC*, *virF*, *yadA* and *ystB*. *Y. kristensenii* isolates harboured the *ystA* gene. Differences between the distribution of virulence factors among *Y. intermedia* STs were not observed (Table 4). The virulence determinants of mobility (*flgA*-*flgN*, *flhA*-*flhE*, *fliA*-*fliT* and *fliZ*), chemotaxis mechanisms (*cheA*, *cheB*, *cheD*, *cheR*, *cheW*, *cheY* and *cheZ*) and genes that encode flagellar motor proteins (*motA* and *motB*) were found in all *Y. enterocolitica* isolates (Supplementary Table S1). 

## 3. Discussion

The contamination of retailed meats with the *Yersinia* species (24%) with *Y. enterocolitica* being predominant was consistent with previous findings [13,27]. The prevalence of *Y. enterocolitica* in beef and pork in our report was higher than that previously reported in Malaysia, Poland, Italy and Egypt [13,28,29,30]. *Yersinia* are psychrotrophic microorganisms, and temperate climatic conditions, including a cold winter season, may enhance the survival of *Yersinia* species in animals and the environment [31]. The unhygienic handling of meat may facilitate the spread of *Yersinia* species, leading to a higher prevalence at the retail level [14]. 

Only pork was found to be contaminated by pathogenic *Y. enterocolitica* 4/O:3 and 2/O:9, while all isolates from beef belonged to non-pathogenic biotype 1A. Pathogenic *Y. enterocolitica* bioserovars (3/O:5,27 and 3/O:9) were identified in cattle [32,33]. Since pathogenic *Y. enterocolitica* (2/O:5,27) was found in bulk milk at dairy farms, and improperly treated pasteurized milk, contaminated with *Y. enterocolitica*, was reported to be the source of yersiniosis outbreak, beef and cattle could be involved in the epidemiology of yersinosis [34,35]. Liang et al. [36] concluded that cattle may act as occasional hosts, while domestic pigs could be the principal reservoir. Pathogenic bioserovars, especially 4/O:3, were often isolated from slaughtered pigs in Europe, being exclusively predominated in pigs from Belgium, Germany and Finland [10,11,12,13,14,16]. The identification of identical genotypes of *Y. enterocolitica* in pigs and retail pork and human isolates confirms their importance in the epidemiology of human yersiniosis [37]. *Y. enterocolitica* 4/O:3 was identified as an important source of sporadic yersiniosis, and *Y. enterocolitica* 2/O:9 was involved in the yersiniosis outbreak in Norway related to undercooked pork meat consumption [18,38]. Since *Y. enterocolitica* 4/O:3 and O:9 were identified at the retail level, this indicates public health implications as contaminated pork could represent a risk for consumers. 

The low recovery of pathogenic *Y. enterocolitica* from foods was linked to the low sensitivity of the conventional detection methods due to the application of ISO 10273:2017 for food testing and the poor ability of *Y. enterocolitica* to compete with background microbiota [39]. *Y. enterocolitica* 4/O:3 counts of 10–10^2^ cfu/g were undetected, while non-pathogenic *Y. enterocolitica* 1A was accurately identified in experimentally contaminated pork cuts [40]. Reported widespread occurrence of *Y. enterocolitica* 1A was in agreement with previous studies [13,29].

The *ail* gene (adhesion and invasion locus) was identified in 3% (6/180) of *Y. enterocolitica*-positive pork samples with qPCR. All *ail*-positive *Y. enterocolitica* isolates belonged to 4/O:3 and 2/O:9 bioserovars. This was in line with previous findings, where the prevalence of pathogenic *Y. enterocolitica* in pork varied from 0% (0/96) in Poland to 10% (46/446) in Germany, detected using a culture method [29,41]. A higher prevalence of the pathogen was recovered when the combination of ISO 10273 and *ail*-based qPCR methods was applied [13,28,29,41,42,43,44]. In 11 isolates of *Y. enterocolitica* 1A, *Y. intermedia* and *Y. kristensenii*, Cts > 35 was identified, which was later confirmed as *ail* negative using WGS. The *ail* gene is crucial for the adhesion and invasion of the pathogen to the host cell and provides serum resistance, thus making it important for the pathogenesis of yersiniosis [45]. The *ail* gene is widely targeted to confirm *Y. enterocolitica* pathogenicity [23]. Previous reports show the sporadic presence of the *ail* gene in *Y. enterocolitica* 1A and other *Yersinia* species in clinical, animal and food samples, raising debates regarding its significance in epidemiology of human yersiniosis [13,23,26,46]. 

The observed high antimicrobial resistance rates in *Y. enterocolitica* 4/O:3 and 2/O:9 pork isolates against ampicillin (100%) were in agreement with the 100% reported in *Y. enterocolitica* 4/O:3 isolates from pigs in Lithuania and Italy [47,48] and *Y. enterocolitica* 1A from foods in China [49]. *Y. enterocolitica* was reported to be naturally resistant to ampicillin and other beta-lactam and streptogramin antibiotics due to the presence of *vat(F)*, *blaA* and *blaB* genes [50,51]. The presence of *blaA* and *blaB* genes in non-pathogenic and pathogenic *Y. enterocolitica*, as well as in other *Yersinia* species, was in line with previous reports (49,50). Additionally, resistance to neomycin, streptomycin, tetracycline, chloramphenicol, cephalosporins and carbapenems was reported, which indicates the potential for the development of antimicrobial resistance in *Y. enterocolitica*. The occurrence of antimicrobial resistance in *Y. enterocolitica* in meats may be attributed to applications in animals; thus, the antimicrobial resistance in *Y. enterocolitica* should be monitored [49,52,53]. 

*ymoA*, *fepD* and *fes* genes were the most common in the *Yersinia* species, while *Y. enterocolitica* harbored *hreP*, *inv*, *myfB*, *myfC*, *sat* and *ymoA* virulence genes. *ymoA* (modulator of the expression of virulence function) was identified in 100% of *Y. enterocolitica* isolated previously [19]. *hreP*, *fepD*, *sat* and *fes* genes were mostly associated with *Y. enterocolitica* 1A [44]. Occasionally, those genes were reported in pathogenic isolates, e.g., *fepD* (enrochelin ABC transporter) in *Y.enterocolitica* 1B/O:8 or *sat* (streptogramin acetyltransferase) in 1B/O:8, 4/O:3 and 3/O:3 bioserovars [19,54,55]. 

In the present work, the presence of *inv*, *ail*, *ystA*, *virF*, *mufA*, *myfB*, *myfC* and *yop* virulon was confirmed in all pathogenic *Y. enterocolitica*, with the exception of *yadA* in the 2/O:9 bioserovar. Plasmid and chromosomal virulence genes are important for the full pathogenicity of *Y. enterocolitica*. *yadA* and *virF* are present in pathogenic strains and located at the virulence plasmid, and are crucial for adherence, the transcriptional activity of *yop* and *yadA* and invasion into the host cell [20,53]. Strains of pathogenic bioserotype 1B/O:8 from pork were reported to be *ail*, *ystA* and virulence plasmid negative due to the apparent loss of pYV [55,56]. 

Aside from chromosomal virulence factors, *inv* (invasion), which is responsible for host cell penetration, was present in all *Y. enterocolitica* [19,49]. *yst* encodes heat stable endotoxins; however, *ystA* is usually confirmed in pathogenic *Y. enterocolitica* and is responsible for diarrhea induction. *ystB* and *ystC* are usually expressed in non-pathogenic *Y. enterocolitica*, but their presence was confirmed in clinical isolates [57,58]. We identified *ystA* in *Y. enterocolitica* 2/O:9 and 4/O:3 and *ystB* in non-pathogenic *Y. enterocolitica*, which corresponds to previous findings [19,49]. The detection of *ail* and *ystB* was proposed for the differentiation of *Y. enterocolitica* 1A and pathogenic 1B/2-5 biotypes by Garzetti et al., 2014 [59]. Our study confirms the correct identification of pathogenic bioserovars using the WGS approach. 

The *myfA* gene promotes the adhesion of the pathogen to enterocytes and was identified in clinical and animal *Y. enterocolitica* 4/O:3 isolates and sporadically in *Y. enterocolitica* 1A isolates. *myfB* and *myfC* are encoded by the *myf* operon and form the fibrillar structure functioning during adhesion, and were associated with pathogenic *Y. enterocolitica* [19,56]. The main differences between *Y. enterocolitica* 1A STs in the present study were related to the distribution of *myfA*, *myfB* and *myfC* genes.

*fepD*, *fes*, *ymoA*, *ystA* and *ystB* virulence genes were confirmed in *Y. kristensenii* and *ymoA* and *ystB* in *Y. intermedia* in the present study. Despite lacking classical virulence markers, with the exception of *ystA* in *Y. kristensenii*, in the present study, other pathogenicity factors may contribute to *Yersinia* virulence. *ystB* of *Y. enterocolitica* 1A was considered as potentially pathogenic, and high similarity between clinical and rodent isolates of *ystB*, *ail* and *inv* fragments was shown [46]. The presence of virulence genes of clinical importance (*ail*, *myfA* and *ystA*) was identified previously in non-pathogenic *Y. enterocolitica* and other *Yersinia*—*Y. kristensenii* and *Y. intermedia* [9,39,46]. 

All characterized *Y. enterocolitica*, *Y. intermedia* and *Y. kristensenii* in the present study shared virulence factors for mobility control, which contribute to invasion, biofilm formation and the secretion system (*flg* and *flh*); genes responsible for chemotaxis mechanisms (*che*); and genes which encode the flagellar motor (*mot*) protein [60,61]. These genes were described in a clinical isolate of *Y. enterocolitica* 4/O:3, and authors stated that a variety of virulence factors could contribute to the successful dissemination of *Y. enterocolitica* 4/O:3 clones globally [56]. 

Out of the STs associated with pathogenic *Y. enterocolitica*, ST18 was reported to correspond to 4/O:3 and ST12 to biotype 2-3/O:9 [62]. ST18 was isolated from clinical cases in Sweden, Germany, New Zealand, France, the United Kingdom and Brazil [26,53,56,63]. ST18 was identified in pigs, dogs and bovine sources [53]. In general, the present study confirmed that *Y. enterocolitica* STs 12 and 18 were associated with pathogenic *Y. enterocolitica* 4/O:3 and 2/O:9 bioserovars. 

Among non-pathogenic *Y. enterocolitica* 1A and *Y. intermedia*, a higher degree of diversity was found with fifteen and seven STs identified, respectively. Since *ail*-negative *Y. enterocolitica* isolates are usually considered as non-pathogenic and rejected without further analysis, the data on the genetic diversity of *Y. enterocolitica* 1A are limited. *Y. enterocolitica* 1A of ST3, ST4, ST137 and ST307 were reported in human cases in England, and ST3 was among the most widespread [26]. This shows that WGS-based techniques may provide new knowledge on the pathogenicity and epidemiology of non-pathogenic and pathogenic *Y. enterocolitica* isolates since the data on the distribution of the MLST types are more informative for understanding the ecology of *Y. enterocolitica* in comparison with routine biotyping and serotyping.

In the present study, the WGS methodology facilitated the identification and evaluation of the virulence characteristics of pathogenic *Y. enterocolitica* strains, and the correct identification of all pathogenic strains of ST18 and ST12 was shown. Additionally, the diversity of *Y. enterocolitica* 1A and the association of the virulence of pathogenic STs with the presence of key virulence determinants in food isolates were shown. 

## 4. Materials and Methods

### 4.1. Sampling

A total of 330 samples of raw pork and beef were collected between 2015 and 2021 from 32 retail outlets in Latvia. Raw pork samples (n = 180) included pork cuts, minced pork and offal (tongue, liver and kidney), and for beef (n = 150), beef cuts were selected in supermarkets from the meats available to consumers. From one to three samples from the same producer were purchased at once, aseptically placed in sample transportation containers and immediately delivered on ice to the laboratory. Investigations were started within 2 h after collection. 

### 4.2. Microbiological Testing of Samples

Samples were investigated according to the ISO 10273:2017 [63]. In brief, 25 g of sample was diluted in 225 mL of Peptone Sorbitol Bile (PSB) broth, which was incubated at 25 °C for 44 h. Enriched broth was placed onto Cefsulodin Irgasan Novobiocin (CIN, Biolife, Milan, Italy) agar with and without 0.5% KOH treatment for 20 s; inoculated agars were incubated at 30 °C for 24 h. Suspicious colonies of *Yersinia* species with red centres and transparent surrounding areas were selected for biochemical confirmation for urea production, sugar fermentation in Triple Sugar Agar (TSI, Biolife) and Decarboxylase Lysine broth (Biolife). After incubation at 30 °C for 24 h, presumed *Yersinia* species colonies were confirmed via matrix-assisted laser desorption/ionization mass spectrometry (MALDI-TOF, Bruker, Bremen, Germany). Cultures of *Yersinia* species were stored in 10% glycerol and Brain Heart Infusion (BHI) media at -80 °C until further investigation. 

### 4.3. Detection of Biotypes and Serogroups of Yersinia Enterocolitica

Biotypes were detected according to Wauters [64], and *Y. enterocolitica* isolates were tested for pyrazimidase and lipase activity, salicine, xylose and trehalose fermentation. Serogroups of *Y. enterocolitica* were detected with commercially available antisera against O:3, O:5, O:8, O:9 and O:27 according to the manufacturer’s instructions (Sifin, Berlin, Germany). 

### 4.4. Detection of Antimicrobial Resistance of Pathogenic Yersinia Enterocolitica

The antimicrobial resistance of *Y. enterocolitica* 4/O:3 and 2/O:9 isolates was detected with broth microdilution method using the EUVSEC panel (TREK Diagnostic Systems Ltd., East Grinstead, UK). The bacterial suspension (0.5 McFarland) in 11 mL of cation-adjusted Mueller-Hinton (MH) broth was used for the inoculation of MIC test panels. Inoculated panels were incubated at 30 °C for 24 h. The antimicrobial resistance was tested against ampicillin (1–64 mg/L), cefotaxime (0.25–4 mg/L), ceftazidime (0.5–8 mg/L), meropenem (0.03–16 mg/L), nalidixic acid (4–128 mg/L), ciprofloxacin (0.015–8 mg/L), tetracycline (2–64 mg/L), colistin (1–16 mg/L), gentamicin (0.5–32 mg/L), trimethoprim (0.25–32 mg/L), sulfamethoxazole (8–1024 mg/L), chloramphenicol (8–128 mg/L), azithromycin (2–64 mg/L) and tigecycline (0.25–8 mg/L). The resistance thresholds were interpreted in accordance with EUCAST [65].

### 4.5. Screening of Pathogenicity of Yersinia Enterocolitica with qPCR

DNA was extracted from fresh cultures using the MagMAX™ Viral/Pathogen II Nucleic Acid Isolation Kit on a KingFisher Flex instrument (ThermoFisher Scientific, Waltham, MA, USA). The *ail* gene of *Yersinia enterocolitica* was targeted for the screening of the pathogenicity of *Yersinia* species. An amount of 2.5 µL was added to 17.5 µL PCR mastermix containing a Luminaris Color Probe qPCR mix (1X) (Thermo Fisher Scientific), 300 nM ail primers (ail-F: 5′-GGT TAT GCA CAA AGC CAT GTA AA-3′, ail-R: 5′-AAA CGA ACC TAT TAC TCC CCA GTT-3′, 93 bp, Bioneer, Daejeon, Korea), 125 nM ail-tmp-probe (5′FAM-AAC CTG AAG TAC CGT TAT GAA CTC GAT GA-BHQ1-3′, 29 bp, Bioneer) and 6.25 µL RNA-free water [66]. The PCR conditions were 50 °C for 2 min, and 95 °C for 10 min, followed by 45 cycles at 95 °C for 10 s and 30 s at 60 °C (QuantStudio 6, ThermoFisher Scientific). 

### 4.6. Genome Sequencing and Analysis

At least one *Y. enterocolitica* isolate from pork and beef recovered from the same meat sample was chosen for WGS (Supplementary Table S3). 

Whole genome sequencing libraries were prepared from the DNA using either a Nextera XT (Illumina, San Diego, CA, USA), Illumina DNA Prep (Illumina) or QIAseq FX (Qiagen, Hilden, Germany) reagent kit. In all library preparation protocols, the final magnetic bead clean-up procedure was modified to select libraries with a longer insert size (approx. 500 bp). The final libraries were sequenced on the MiSeq instrument (Illumina) to yield 2 × 250 or 2 × 300 bp paired-end reads.

The Trimmomatic v0.38 software was used to remove sequencing adapters and low-quality bases from the raw reads [67]. The trimmed reads were then de novo assembled by the SPAdes assembler v3.14.0 [68]. Bacterial species assignment and the presence of contamination were verified by the taxonomic classification of reads against the MiniKraken (v1_8GB_201904) database using Kraken v2.0.8 [69]. Genomes that appeared contaminated, too fragmented (N50 < 10 kb) or were of inappropriate length were excluded from further analysis.

The presence of virulence trait-encoding genes was determined using a BLAST-based approach and gene reference sequences from the Virulence Factor Database [70]. All genes from the *Yersina* section of VFDB were included, and a few others were added (see Supplementary Table S2). Any gene was considered to be present in the genome if at least 70% of its length was matched with at least 70% nucleotide identity in the contigs (except for *ystA* and *ystB*, for which 80% minimum identity was required). Based on the presence of *ail*, *inv*, *ystA* and *ystB* virulence determinants, *Y. enterocolitica* strains were grouped into non-virulent or virulent biotypes (1A or 1B/2-5, respectively), as described by Garzetti et al. (2014) [59].

To explore the diversity of *Yersinia* genomes, an allele-by-allele approach was used. Raw reads were uploaded to Enterobase, where multi-locus sequence typing (MLST) and core genome MLST (cgMLST) were performed [71]. The McNally seven-gene MLST scheme was used [72]. Genomic relationships based on cgMLST profiles were calculated with the MSTree V2 algorithm and visualized in GrapeTree [73]. 

### 4.7. Data Analysis

The significance (*p* < 0.05) of differences in the prevalence of *Yersinia* spp. and *Y. enterocolitica* in different meat categories was calculated using the Chi-square test. 

## 5. Conclusions

Higher genetic diversity was observed for *Y. enterocolitica* 1A and other *Yersinia* species in comparison to pathogenic *Y. enterocolitica* 4/O:3 and 2/O:9. Virulence markers may represent the unique virulence properties of each ST, providing important information on the significance of pathogenic *Y. enterocolitica* and non-pathogenic *Yersinia* species in the epidemiology of yersiniosis. The WGS analysis of *Y. enterocolitica* showed the accurate identification of non-pathogenic 1A and pathogenic 1B/2-5 biotypes. Retail pork contaminated with pathogenic *Y. enterocolitica* represents public health concerns, since pathogenic *Y. enterocolitica* harbours key virulence factors for the induction of infection in humans. 

## Figures and Tables

**Table 1:** Prevalence of *Yersinia* species in meats at the retail market.

Meat Category	Sample Category	No. of Sample	*Yersinia* spp.	*Y. enterocolitica*	*Y. intermedia*	*Y. kristensenii*	*Y. massiliensis*	*Y. frederiksenii*	*Y. molaretti*
			**No. of Positive Samples (%)**						
Pork	Pork cuts	160	42 (26)	36 (23)	4 (3)	1 (1)	1 (1)	2 (1)	0 (0)
	Minced pork	9	7 (78)	3 (33)	3 (33)	2 (22)	1 (11)	0 (0)	0 (0)
	Offal	11	6 (55)	5 (45)	3 (28)	1 (9)	0 (0)	1 (9)	1 (9)
Beef	Beef cuts	150	24 (16)	19 (13)	6 (4)	0 (0)	0 (0)	0 (0)	0 (0)
Total		330	79 (24)	63 (19) ^b^	16 (5)	4 (1)	2 (1)	3 (1)	1 (1)

^b^—prevalence of *Y. enterocolitica* in meat samples was higher (*p* < 0.05) than the prevalence of other *Yersinia* species.

**Table 2:** Antimicrobial resistance in *Yersinia enterocolitica* 4/O:3 and 2/O:9 isolates.

Agent	MIC Resistance Breakpoint (mg/L)	Identified MIC (mg/L) Range	No. of Resistant Isolates (%)
Ampicillin	8	16–64	6 (100)
Azithromycin	NA	<2–4	NA
Cefotaxime	2	<0.25	0 (0)
Ceftazidime	4	<0.5	0 (0)
Ciprofloxacin	0.5	<0.015	0 (0)
Chloramphenicol	8	<8	0 (0)
Colistin	2	<1	0 (0)
Gentamicin	2	<0.5	0 (0)
Meropenem	8	<0.03	0 (0)
Nalidixic acid	NA	<4	NA
Tetracycline	4	<2	0 (0)
Tigecycline	0.5	<0.25	0 (0)
Trimetoprim	4	0.5–2	0 (0)
Sulfametoxazole	NA	<8–16	NA

NA—resistance breakpoints are not established.

**Table 3:** Sequence types (STs) of *Yersinia* isolates found in meat samples.

*Y. enterocolitica*		*Y. frederiksenii*	*Y. intermedia*	*Y. kristensenii*
1A	1B/2-5			
**ST (No. of Isolates)**				
3 (2)	12 (1)	685 (1) ^a^	68 (1)	687 (1) ^a^
4 (1)	18 (4)		140 (1)	
137 (2)			680 (1) ^a^	
147 (1)			681 (1) ^a^	
163 (1)			682 (1) ^a^	
219 (1)			683 (1) ^a^	
278 (1)			686 (1) ^a^	
307 (1)				
317 (1)				
389 (1)				
455 (1)				
684 (1)				
688 (1)				

ST—sequence type; ^a^ novel STs according to Enterobase.

**Table 4:** Distribution of major virulence determinants in *Yersinia* species isolated from meats.

*Yersinia* Species	ST	Virulence Genes															
		*ail*	*fepD*	*fes*	*hreP*	*inv*	*myfA*	*myfB*	*myfC*	*sat*	*virF*	*yadA*	*ymoA*	*ystA*	*ystB*	*blaA*	*blaB*
*Y. enterocolitica*	12	1	1	1	1	1	1	1	1	1	1	0	1	1	0	1	1
	18	1	0	0	1	1	1	1	1	1	1	1	1	1	0	1	1
	3	0	1	1	1	1	0	1	1	1	0	0	1	0	1	1	1
	4	0	1	1	1	1	0	1	1	1	0	0	1	0	1	1	1
	137	0	1	1	1	1	0	1	1	1	0	0	1	0	1	1	1
	147	0	1	1	1	1	0	1	1	1	0	0	1	0	1	1	1
	163	0	1	1	1	1	0	1	1	1	0	0	1	0	1	1	1
	219	0	1	1	1	1	0	1	1	1	0	0	1	0	1	1	1
	278	0	1	1	1	1	0	1	1	1	0	0	1	0	1	1	1
	307	0	1	1	1	1	0	1	1	1	0	0	1	0	1	1	1
	317	0	1	1	1	1	1	1	1	1	0	0	1	0	1	1	1
	389	0	1	1	1	1	1	1	1	1	0	0	1	0	1	1	1
	455	0	1	1	1	1	0	1	1	1	0	0	1	0	1	1	1
	684	0	1	1	1	1	1	1	1	1	0	0	1	0	1	1	1
	688	0	0	0	1	1	1	1	1	1	0	0	1	0	1	1	1
*Y. frederiksenii*	685	0	1	0	0	0	0	0	0	0	0	0	1	0	0	1	1
*Y. intermedia*	All STs	0	1	1	0	0	0	0	0	1	0	0	1	0	0	1	1
*Y. kristensenii*	687		1	1	1	0	0	0	0	0	0	0	1	1	0	1	1

ST—sequence type; 0—virulence gene was not identified; 1—virulence gene was identified.

## Data Availability

Raw sequence reads have been deposited in the European Nucleotide Archive under the study accession number PRJEB49068.

## Supplementary Materials

The following supporting information can be downloaded at: https://www.mdpi.com/article/10.3390/pathogens11010037/s1, Table S1: Virulence factors of *Yersinia* species; Table S2: List of virulence gene references used in the analysis; Table S3: *Yersinia* species isolates selected for whole genome sequencing (WGS) analysis.

## References

[1] Bottone E.J. (2015). *Yersinia enterocolitica*: Revisitation of enduring human pathogen. Clin. Microbiol. Newsl..

[2] Hammerl J.A., Barac A., Erben P., Fuhrmann J., Gadicherla A., Kumsteller F., Lauckner A., Müller F., Hertwig S. (2021). Properties of two broad host range phages of *Yersinia enterocolitica* isolated from wild animals. Int. J. Mol. Sci..

[3] Rosner B.M., Stark K., Werber D. (2010). Epidemiology of reported *Yersinia enterocolitica* infections in Germany, 2001–2008. BMC Public Health.

[4] Le Guern A.S., Martin L., Savin C., Carniel E. (2016). Yersiniosis in France: Overview and potential sources of infection. Int. J. Infect. Dis..

[5] European Food Safety Authority and European Centre for Disease Prevention and Control (EFSA and CDC) (2021). The European Union One Health 2019 Zoonoses Report. EFSA J..

[6] Cornelis G.R., Boland A.P., Boyn A.P., Geuijen C., Iriarte M., Neyt C., Sory M.P., Stainier I. (1998). The virulence plasmid of *Yersinia*, an antihost genome. Microbiol. Mol. Biol. Rev..

[7] Howard S.L., Gaunt M.W., Hinds J., Witney A.A., Stabler R., Wren B.W. (2006). Application of comparative phylogenomics to study the evolution of *Yesinia enterocolitica* and to identify genetic differences relating to pathogenicity. J. Bacteriol..

[8] Tennant S.M., Grant T.H., Robins-Browne R.M. (2003). Pathogenicity of *Yersinia enterocolitica* biotype 1A. FEMS Immunol. Med. Microbiol..

[9] Murros A., Säde E., Johansson P., Korkeala H., Fredriksson-Ahomaa M., Björkroth J. (2016). Characterization of European *Yersinia enterocolitica* 1A strains using restriction fragment length polymorphism and multilocus sequence analysis. Lett. Appl. Microbiol..

[10] Laukkanen-Ninious R., Fredriksson-Ahomaa M., Korkeala H. (2014). Enteropathogenic *Yersinia* in the pork production chain: Challenges for control. Compr. Rev. Food Sci. Food Saf..

[11] Bari M.L., Hossain H.A., Isshiki K., Ukuku D. (2011). Behavior of *Yersinia enterocolitica* in foods. J. Pathog..

[12] Van Damme I., Berkvens D., Vanantwerpen G., Baré J., Houf K., Wauters G., De Zutter L. (2015). Contamination of freshly slaughtered pig carcasses with enteropathogenic *Yersinia* spp.: Distribution, quantification and identification of risk factors. Int. J. Food Microbiol..

[13] Bonardi S., Paris A., Bassi L., Salmi F., Bacci C., Riboldi E., Boni E., D’Incau M., Tagliabue S., Brindani F. (2010). Detection, semiquantitative enumeration, and antimicrobial susceptibility of *Yersinia enterocolitica* in pork and chicken meats in Italy. J. Food Prot..

[14] Fredriksson-Ahomaa M., Koch U., Bucher M., Stolle A. (2004). Different genotypes of *Yersinia enterocolitica* 4/O:3 strains widely distributed in butchers shops in the Munich area. Int. J. Food Microbiol..

[15] Lorencova A., Slany M. (2016). Prevalence of pathogenic *Yersinia enterocolitica* in minced meat, pig tongues and hearts at the retail level in the Czech Republic detected by real time PCR. Potravinarstvo.

[16] Laukkanen-Ninios R., Fredriksson-Ahomaa M., Maijala R., Korkeala H. (2014). High prevalence of pathogenic *Yersinia enterocolitica* in pig cheeks. Food Microbiol..

[17] Lucero-Estrada C.S.M., Favier G.I., Escudero M.E. (2020). An overview of *Yersinia enterocolitica* and related species in samples of different origin from San Luis, Argentina. Food Microbiol..

[18] Guillier L., Fravalo P., Leclercq A., Thébault A., Kooh P., Cadavez V., Gonzales-Barron U. (2021). Risk factors for sporadic *Yersinia enterocolitica* infections: A systematic review and meta-analysis. Microb. Risk Anal..

[19] Morka K., Wałecka-Zacharska E., Schubert J., Dudek B., Woźniak-Biel A., Kuczkowski M., Wieliczko A., Bystrón J., Bania J., Bugla-Płoskońska G. (2021). Genetic diversity and distribution of virulence-associated genes in *Y. enterocolitica* and *Y. enterocolitica*-like isolates from humans and animals in Poland. Pathogens.

[20] Bancerz-Kisiel A., Pieczywek M., Łada P., Szweda W. (2018). The most important virulence markers of *Yersinia enterocolitica* and their role during infection. Genes.

[21] (2015). ISO 18867:2015; Microbiology of the Food Chain–Polymerase Chain Reaction–Detection of Pathogenic Yersinia enterocolitica and Yersinia pseudotuberculosis.

[22] Imori P.F.M., Passaglia J., Souza R.A., Rocha L.B., Falcao J.P. (2017). Virulence-related genes, adhesion and invasion of some *Yersinia enterocolitica*-like strains suggests its pathogenic potential. Microb. Pathog..

[23] Kraushaar B., Dieckmann R., Wittwer M., Knabner D., Konietzny A., Mäde D., Strauch E. (2011). Characterization of a *Yersinia enterocolitica* biotype 1A strain harbouring an *ail* gene. J. Appl. Microb..

[24] Ashton P.M., Nair S., Peters T.M., Bale J.A., Powell D.G., Painset A., Tewolde R., Schaefer U., Jenkins C., Dallman T.J. (2016). Identification of *Salmonella* for public health surveillance using whole genome sequencing. PeerJ.

[25] Inns T., Flanagan S., Greig D.R., Jenkins C., Seddon K., Chin T., Cartwright J. (2018). First use of whole-genome sequencing to investigate a cluster of *Yersinia enterocolitica*, Liverpool, United Kingdom, 2017. J. Med. Microbiol..

[26] Hunter E., Greig D.R., Schaefer U., Wright M.J., Dallman T.J., McNally A., Jenkins C. (2018). Identification and typing of *Yersinia enterocolitica* and *Yersinia pseudotuberculosis* isolated from human clinical specimens in England between 2004 and 2018. J. Med. Microbiol..

[27] Özdemir F., Arslan S. (2015). Genotypic and phenotypic virulence characteristics and antimicrobial resistance of *Yersinia* spp. isolated from meat and milk products. J. Food Sci..

[28] Tan L.K., Ooi P.T., Thong K.L. (2014). Prevalence of *Yersinia enterocolitica* from food and pigs in selected states of Malaysia. Food Control.

[29] Zadernowska A., Chajęcka-Wierzchowska W. (2017). Prevalence, biofilm formation and virulence markers of *Salmonella* sp. and *Yersinia enterocolitica* in food of animal origin in Poland. LWT-Food Sci. Technol..

[30] Younis G., Mady M., Awad A. (2019). *Yersinia enterocolitica*: Prevalence, virulence, and antimicrobial resistance from retail and processed meat in Egypt. Vet. World.

[31] Syczyło K., Platt-Samoraj A., Bancerz-Kisiel A., Szczerba-Turek A., Pajdak-Czaus J., Łabuć S., Procajło Z., Socha P., Chuzhebayeva G., Szweda W. (2018). The prevalence of *Yersinia enterocolitica* in game animals in Poland. PLoS ONE.

[32] McNally A., Cheasty T., Fearnley C., Dalziel R.W., Paiba G.A., Manning G., Newell D.G. (2004). Comparison of the biotypes of *Yersinia enterocolitica* isolated from pigs, cattle and sheep at slaughter and from humans with yersiniosis in Great Britain during 1999–2000. Lett. Appl. Microbiol..

[33] Bonardi S., Paris A., Bacci C., D’Incau M., Ferroni L., Brindani F. (2007). Detection and characterization of *Yersinia enterocolitica* from pigs and cattle. Vet. Res. Commun..

[34] Longenberger A.H., Gronostaj M.P., Yee G.Y., Johnson L.M., Lando J.F., Voorhees R.E., Waller K., Weltman A.C., Moll M., Lyss S.B. (2014). *Yersinia enterocolitica* infections associated with improperly pasteurized milk products: Southwest Pennsylvania, March-August, 2011. Epidemiol. Infect..

[35] Bonardi S., Le Guern A.S., Savin C., Pupillo G., Bolzoni L., Cavalca M., Pongolini S. (2018). Detection, virulence and antimicrobial resistance of *Yersinia enterocolitica* in pulk tank milk in Italy. Int. Dairy J..

[36] Liang J., Duan R., Xia S., Hao Q., Yang J., Xiao Y., Qui H., Shi G., Wand S., Gu W. (2015). Ecology and geographic distribution of *Yersinia enterocolitica* among livestock and wildlife in China. Vet. Microbiol..

[37] Fredriksson-Ahomaa M., Stolle A., Korkeala H. (2006). Molecular epidemiology of *Yersinia enterocolitica* infections. FEMS Immunol. Med. Microbiol..

[38] Grahek-Ogden D., Schimmer B., Cudjoe K.S., Nygård K., Kapperud G. (2007). Outbreak of *Yersinia enterocolitica* serogroup O:9 infection and processed pork, Norway. Emerg. Infect. Dis..

[39] Van Damme I., Berkvens D., Botteldoorn N., Dierick K., Wits J., Pochet B., De Zutter L. (2013). Evaluation of the ISO 10273:2003 method for the isolation of human pathogenic *Yersinia enterocolitica* from pig carcasses and minced meat. Food Microbiol..

[40] Peruzy M.F., Aponte M., Proroga Y.T.R., Capuano F., Cristiano D., Delibato E., Houf K., Murru N. (2020). *Yersinia enterocolitica* detection in pork products: Evaluation of isolation protocols. Food Microbiol..

[41] Messelhäusser U., Kämpf P., Colditz J., Bauer H., Schreiner H., Höller C., Busch U. (2011). Qualitative and quantitative detection of human pathogenic *Yersinia enterocolitica* in different food matrices at retail level in Bavaria. Foodborne Pathog. Dis..

[42] Terentjeva M., Bērziņš A. (2013). Prevalence of *Yersinia enterocolitica* 4/O:3 in raw pork at retail market in Latvia. Arch. Lebensmittelhyg.

[43] Shoaib M., Shehzad A., Raza H., Niazi S., Khan I.M., Akhtar W., Safdar W., Wand Z. (2019). A comprehensive review of the prevalence, pathogenesis and detection of *Yersinia enterocolitica*. RSC Adv..

[44] Wang J., Liu M., Wang H., Wu Q., Xu T., Ma G., Zhong Y., Zhang J., Chen M., Zue L. (2021). Occurrence, molecular characterization, and antimicrobial susceptibility of *Yersinia enterocolitica* isolated from retail food samples in China. LWT.

[45] Felek S., Krukonis E.S. (2009). The *Yersinia pestis* Ail protein mediates binding and Yop delivery to host cells required for plague virulence. Infect. Immun..

[46] Platt-Samoraj A., Kończyk-Kmiecik K., Bakuła T. (2021). Occurrence and genetic correlations of *Yersinia* spp. isolated from commensal rodents in Northeastern Poland. Pathogens.

[47] Novoslavskij A., Kudirkiené E., Marcinkuté A., Bajoriūniené A., Korkeala H., Malakauskas M. (2013). Genetic diversity and antimicrobial resistance of *Yersinia enterocolitica* isolated from pigs and humans in Lithuania. J. Sci. Food Agric..

[48] Bonardi S., Bruini I., D’Incau M., VanDamme I., Carniel E., Brémont S., Cavallini P., Tagliabue S., Brindani F. (2016). Detection, seroprevalence and antimicrobial resistance of *Yersinia enterocolitica* and *Yersinia pseudotuberculosis* in pig tonsils in Northern Italy. Int. J. Food Microbiol..

[49] Ye Q., Wu Q., Hu H., Zhang J., Huang H. (2016). Prevalence and characterization of *Yersinia enterocolitica* isolated from retail foods in China. Food Control.

[50] Bonke R., Wacheck S., Stüber E., Meyer C., Märlbauer E., Fredriksson-Ahomaa M. (2011). Antimicrobial susceptibility and distribution of β-lactamase A(*blaA*) and β-lactamase B (*blaB*) genes in enteropathogenic *Yersinia* species. Microb. Drug Resist..

[51] Seoane A., García Lobo J.M. (2000). Identification of a streptogramin a acetyltransferase gene in the chromosome of *Yersinia enterocolitica*. Antimicrob. Agents Chemother..

[52] Bolton D.J., Ivory C., McDowell D. (2013). A small study of *Yersinia enterocolitica* in pigs from birth to carcass and characterization of porcine and human strains. Food Control.

[53] Karlsson P.A., Tano E., Jernberg C., Hickman R.A., Guy L., Järhult J.D., Wang H. (2021). Molecular characterization of multidrug-resistant *Yersinia enterocolitica* from foodborne outbreaks in Sweden. Front. Microbiol..

[54] Bhagat N., Virgi J.S. (2007). Yersinia enterocolitica 1A correlates with clonal groups and not the source of isolation. FEMS Microbiol. Lett..

[55] Thong K.L., Tan L.K., Ooi P.T. (2018). Genetic diversity, virulotyping and antimicrobial resistance susceptibility of Yersinia enterocolitica isolated from pigs and porcine products in Malaysia. J. Sci. Food Agric..

[56] Rusak L.A., Junqueira R.M., Hofer E., Vallim D.C., Asensi M.D. (2017). Next-generation sequencing virulome analysis of a *Yersinia enterecolitica* subsp. *palearctica* bioserotype 4/O:3 ST18 isolated from human blood in Brazil. Braz J. Infect. Dis..

[57] Singh I., Virdi J.S. (2004). Production of Yersinia stable toxin (YST) and distribution of *yst* genes in biotype 1A strains of *Yersinia enterocolitica*. J. Med. Microb..

[58] Zheng H., Sun Y., Mao Z., Jiang B. (2008). Investigation of virulence genes in clinical isolates of *Yersinia enterocolitica*. FEMS Immunol. Med. Microbiol..

[59] Garzetti D., Susen R., Fruth A., Tietze E., Heesemann J., Rakin A. (2014). A molecular scheme for *Yersinia enterocolitica* patho-serotyping derived from genome-wide analysis. Int. J. Med. Microbiol..

[60] Bren A., Eisenbach M. (2000). How signals are heard during bacterial chemotaxis: Protein-protein interactions in sensory signal propagation. J. Bacteriol..

[61] Kim T.J., Young B.M., Young G.M. (2008). Effect of flagellar mutations on *Yersinia enterocolitica* biofilm formation. Appl. Environ. Microbiol..

[62] Strydom H., Wand J., Paine S., Dyet K., Cullen K., Wright J. (2019). Evaluating sub-typing methods for pathogenic *Yersinia enterocolitica* to support outbreak investigations in New Zealand. Epidemiol. Infect..

[63] (2017). ISO 10273:2017; Microbiology of Food and Animal Feed. Horizontal Method for the Detection of Presumably Pathogenic Yersinia enterocolitica.

[64] Wauters G., Kandolo K., Janssens M. (1987). Revised biogrouping scheme of *Yersinia enterocolitica*. Contrib. Microbiol. Immunol..

[65] European Committee on Antimicrobial Susceptibility Testing (EUCAST) Breakpoints Tables for Interpretation of MICs and Zones Diameters. Version 11.0. https://www.eucast.org/fileadmin/src/media/PDFs/EUCAST_files/Breakpoint_tables/v_11.0_Breakpoint_Tables.pdf.

[66] Mäde D., Reiting R., Strauch E., Ketteritzsch K., Wicke A. (2008). A real-time PCR for detection of pathogenic *Yersinia enterocolitica* in food combined with an universal internal amplification control system. J. Verbr. Lebensm.

[67] Bolger A.M., Lohse M., Usadel B. (2014). Trimmomatic: A flexible trimmer for Illumina sequence data. Bioinformatics.

[68] Prjibelski A.D., Puglia G.D., Antipov D., Bushmanova E., Giordano D., Mikheenko A., Vitale D., Lapidus A. (2020). Extending rnaSPAdes functionality for hybrid transcriptome assembly. BMC Bioinform..

[69] Wood D.E., Lu J., Langmead B. (2019). Improved metagenomic analysis with Kraken 2. Genome Biol..

[70] Liu B., Zheng D., Jin Q., Chen L., Yang J. (2019). VFDB 2019: A comparative pathogenomic platform with an interactive web interface. Nucleic Acids Res..

[71] Zhou Z., Alikhan N.F., Mohamed K., Achtman M., the Agama Study Group (2020). The EnteroBase user’s guide, with case studies on *Salmonella* transmissions, *Yersinia pestis* phylogeny and *Escherichia* core genomic diversity. Genome Res..

[72] Hall M., Chattaway M.A., Reuter S., Savin C., Strauch E., Carniel E., Connor T., Van Damme I., Rajakaruna L., Rajendram D. (2015). Use of whole-genus genome sequence data to develop a multilocus sequence typing tool that accurately identifies Yersinia isolates to the species and subspecies levels. J. Clin. Microbiol..

[73] Zhou Z., Alikhan N.F., Sergeant M.J., Luhmann N., Vaz C., Francisco A.P., Carrico J.A., Achtman M. (2018). GrapeTree: Visualization of core genomic relationships among 100,000 bacterial pathogens. Genome Res..

